# Crystal structure and Hirshfeld surface analysis of one-dimensional copper(II) coordination polymer incorporating succinate and tetra­methyl­ethylene­diamine ligands

**DOI:** 10.1107/S2056989020007227

**Published:** 2020-06-09

**Authors:** Adnan M. Qadir, Sevgi Kansiz, Necmi Dege, Georgina M. Rosair, Turganbay S. Iskenderov

**Affiliations:** a Department of Chemistry, College of Science, Salahaddin University, Erbil, 44001, Iraq; b Department of Fundamental Sciences, Faculty of Engineering, Samsun University, Samsun, 55420, Turkey; c Department of Physics, Faculty of Arts and Sciences, Ondokuz Mayıs University, Samsun, 55139, Turkey; d Institute of Chemical Sciences, School of Engineering & Physical Sciences, Heriot-Watt University, Edinburgh, EH14 4AS, UK; e Department of Chemistry, National Taras Shevchenko University of Kyiv, 64, Vladimirska Str., Kiev 01601, Ukraine

**Keywords:** crystal structure, copper(II), polymer, tetra­methyl­ethylenedi­amine, Hirshfeld surface

## Abstract

The reaction of copper nitrate with succinic acid (succH) and *N*,*N*,*N*′,*N*′-tetra­methyl­ethylenedi­amine (TMEDA) in basic solution produces the complex *catena*-poly[[[(*N*,*N*,*N*′,*N*′-tetra­methyl­ethylenedi­amine-κ^2^ *N*,*N*′)copper(II)]-μ-succinato-κ^2^ *O* ^1^:*O* ^4^] tetra­hydrate], {[Cu(C_4_H_4_O_4_)(C_6_H_16_N_2_)]·4H_2_O}_*n*_ or {[Cu(succ)(tmeda)]·4H_2_O}_*n*_. Each carboxyl­ate group of the succinate ligand coordinates to a Cu^II^ atom in a monodentate fashion, giving rise to a distorted square-planar geometry. The succinate ligands bridge the Cu^II^ centres, forming one-dimensional polymeric chains. Hydrogen bonds between the ligands and water mol­ecules link these chains into sheets that lie parallel to the *ac* plane. Hirshfeld surface analysis, *d*_norm_ and two-dimensional fingerprint plots were examined to verify the contributions of the different inter­molecular contacts within the supra­molecular structure.

## Abstract 2

The title compound, {[Cu(succ)(tmeda)]·4H_2_O}_*n*_, consists of one-dimensional polymeric chains in which the central metal atom is coordinated in a distorted square-planar geometry by one oxygen atom each from two succ ligands and two TMEDA ligand nitro­gen atoms.

## Chemical context   

Coordination polymers are a key area of development in supra­molecular chemistry. Aliphatic saturated di­carboxyl­ates are versatile linkage ligands for construction of supra­molecular frameworks. These possess conformational freedom and coordinating ability owing to the single carbon chain. Aliphatic di­carboxyl­ate anions exhibit different coordination modes such as uni-bidentate, bis-monodentate, bis-bidentate, tridentate or tetra­dentate, linking metal atoms into 1-D coordination polymers, 2-D layers or 3-D networks. Copper(II) carboxyl­ate complexes are known to possess various biological activities including anti­fungal (Melník *et al.*, 1982 ▸), anti­bacterial (Mojumdar *et al.*, 2005 ▸), anti­viral and cytotoxic activities (Ranford *et al.*, 1993 ▸). Copper(II) is present at the active site some of proteins. The proteins containing copper(II) display biological functions such as electron transfer, di­oxy­gen transfer, oxygenation, reduction, oxidation and disproportionation (Mukherjee, 2003 ▸). In this work, the synthesis, single crystal structure and Hirshfeld surface analysis of a copper(II) complex involving *N*,*N*,*N*′,*N*′-tetra­methyl­ethylenedi­amine and succinate ligands are reported.
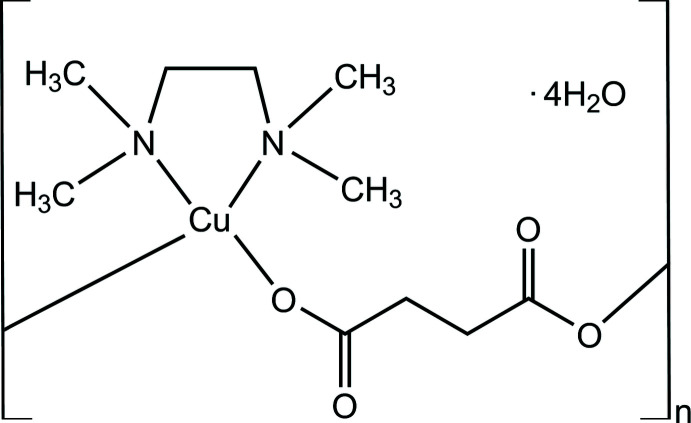


## Structural commentary   

**Figure 1:**
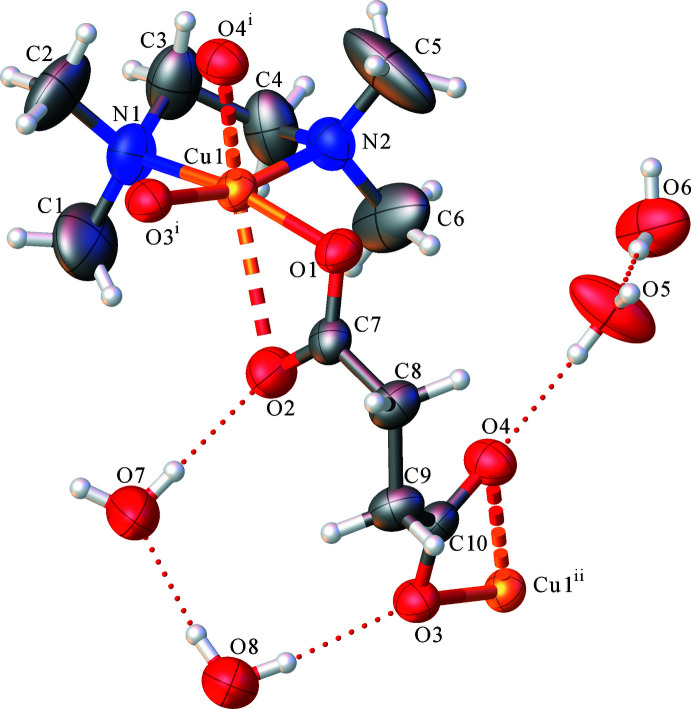
Perspective view of {[Cu(succ)(tmeda)]·4H_2_O}_*n*_, with the atom labelling. Displacement ellipsoids are drawn at the 50% probability level [symmetry codes: (i) −1 + *x*, *y*, *z*; (ii) 1 + *x*, *y*, *z*].

**Figure 2:**
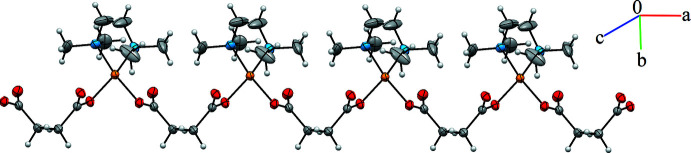
Ellipsoid plot (50%) of a section of the polymeric chain of {[Cu(succ)(tmeda)]·4H_2_O}_*n*_.

The asymmetric unit of the title compound (1) is illustrated in Fig. 1 ▸. In the complex {[Cu(succ)_2_(tmeda)]·4H_2_O}_*n*_, the central metal atom has distorted square-planar geometry with one oxygen atom each from two succ ligands and two TMEDA ligand nitro­gen atoms (Figs. 2 ▸ and 3 ▸). There are two longer axial Cu⋯O contacts of 2.590 (2) and 2.432 (2) Å. In the square-plane, the Cu—O and Cu—N bond lengths are in the range 1.964 (2)–2.038 (2) Å (Table 1 ▸). The structural parameters in the TMEDA ligand, *i.e*. the Cu—N bond lengths, are in agreement with those reported for the [Cu_3_(PyDHA-2H)(tmeda)_3_](ClO_4_)_2_ complex (PyDHA = pyridine-2,6-di­hydroxamic acid) by Gumienna-Kontecka *et al.* (2013 ▸). Similar geometric parameters have also been reported for {[Cu(succ)(deed)]·4H_2_O}_*n*_ [Cu—O: 2.123 (8)–2.142 (8) Å deed = *N*,*N*-diethylethylenediamine; Şen *et al.*, 2017 ▸] and [Cu_2_(C_4_H_4_O_4_)_2_(C_12_H_12_N_2_)]_*n*_, [Cu—O: 1.955 (4)–1.983 (5) Å; González Garmendia *et al.*, 2009 ▸]. Selected bond lengths and angles are given in Table 1 ▸. The succinate ligands bridge the Cu^II^ centres, forming one-dimensional polymeric chains.

## Supra­molecular features   

**Figure 3:**
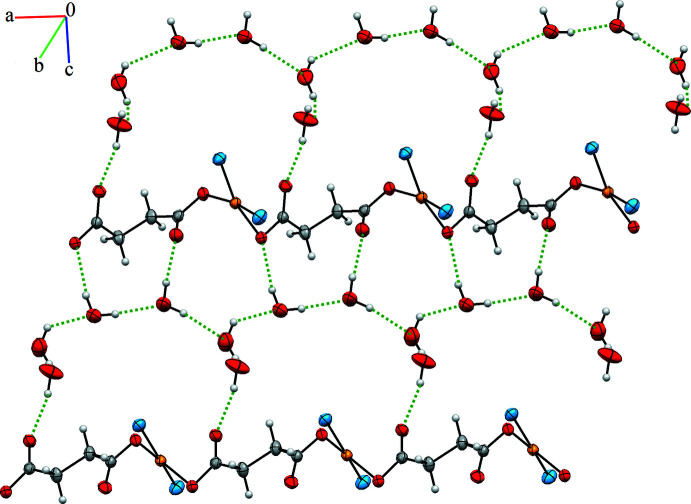
The two-dimensional layered structure of {[Cu(succ)(tmeda)]·4H_2_O}_*n*_. For clarity, the TMEDA ligands are shown only by their N atoms.

In the asymmetric unit of the title complex, there are O5—H5*H*⋯O4, O6—H6*G*⋯O5, O7—H7*A*⋯O2, O8—H8*C*⋯O3 and O8—H8*D*⋯O7 hydrogen-bonding inter­actions, which act to stabilize the crystal packing. The crystal packing (Fig. 3 ▸) also features symmetry-related inter­molecular hydrogen bonds (O7—H7*B*⋯O6^ii^, O5—H5*G*⋯O1^iii^ and O6—H6*H*⋯O8^iv^; symmetry codes as in Table 2 ▸), linking the one-dimensional polymeric chains into sheets that lie parallel to the *ac* plane.

## Database survey   

A search of the Cambridge Structural Database (CSD, version 5.40, update of February 2019; Groom *et al.*, 2016 ▸) for the title complex gave four hits: aqua­(cyclo­butane-1,1-di­carboxyl­ato)(*N*,*N*,*N*′,*N*′-tetra­methyl­ethylenedi­amine)­copper(II) mono­hydrate (CBXECU; Pajunen & Pajunen, 1979*a* ▸), bis­(μ_2_-glutarato)bis­[(*N*,*N*,*N*′,*N*′-tetra­ethyl­ethylenedi­amine)­cop­per(II)] (GLUECU; Pajunen & Pajunen, 1979*b* ▸), [*N*-(2-oxybenzyl­idene)valinato](*N*,*N*,*N*′,*N*′-tetra­methyl­ethane-1,2-di­amine)­copper(II) (UZAPES; Lakshmi *et al.*, 2016 ▸) and (*N*,*N*,*N*′,*N*′′,*N*′′-penta­methyl­diethylenetri­amine)(l-valinato)copper(II) perchlorate (VEGRUU; Murakami & Kita, 1998 ▸). The Cu—N bond lengths range from 1.941 to 2.415 Å. When these bond lengths are compared with the title complex, the Cu—N bond lengths [2.024 (2)–2.038 (2) Å] fall within these limits.

## Hirshfeld surface analysis   

**Figure 4:**
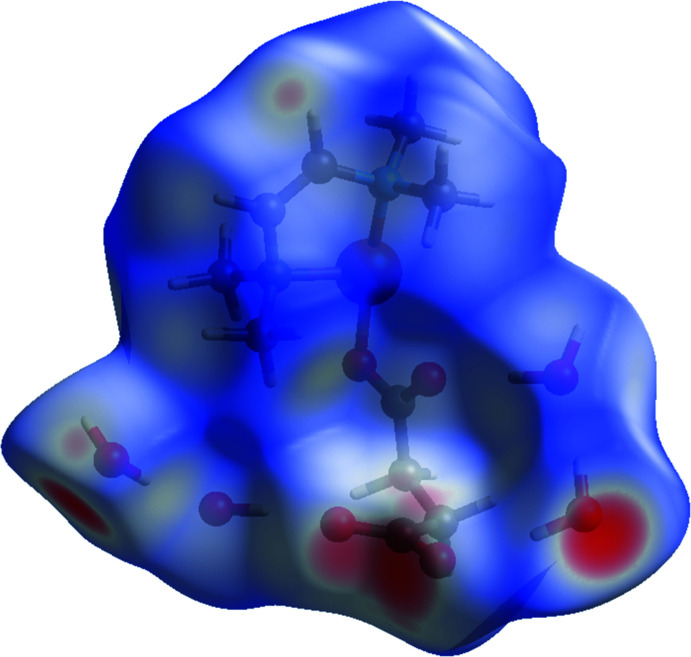
Hirshfeld surface mapped over *d_norm_* for {[Cu(succ)(tmeda)]·4H_2_O}_*n*_.

**Figure 5:**
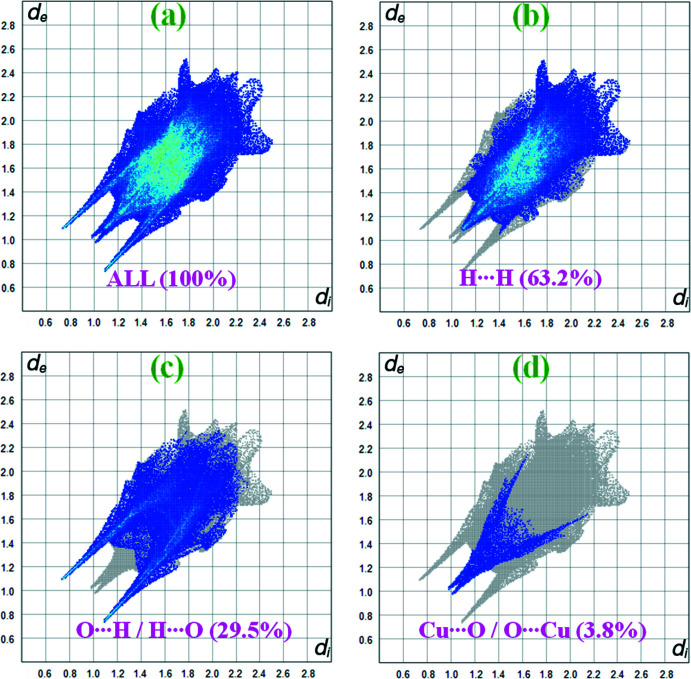
The two-dimensional fingerprint plots for {[Cu(succ)(tmeda)]·4H_2_O}_*n*_ showing the main inter­actions and their percentage contributions (*d*_i_ is the closest inter­nal distance from a given point on the Hirshfeld surface and *d*_e_ is the closest external contact).

The Hirshfeld surface analysis (Spackman & Jayatilaka, 2009 ▸) and the associated two-dimensional fingerprint plots were performed with *CrystalExplorer17* (Turner *et al.*, 2017 ▸). Hirshfeld surface analysis enables the visualization of inter­molecular inter­actions by different colours and colour intensity, representing short or long contacts and indicating the relative strength of the inter­actions. Fig. 4 ▸ shows the Hirshfeld surface mapped over *d*_norm_ (–0.629 to 1.578 a.u.). The overall two-dimensional fingerprint plot for the title complex and those delineated into H⋯H, O⋯H/H⋯O and Cu⋯O/O⋯Cu contacts are illustrated in Fig. 5 ▸. The percentage contributions from the different inter-atomic contacts to the Hirshfeld surface are as follows: H⋯H (63.2%), O⋯H/H⋯O (29.5%) and Cu⋯O/O⋯Cu (3.8%). The percentage contributions for other inter­molecular contacts amount to less than 3% of the Hirshfeld surface mapping.

## Synthesis and crystallization   

An aqueous solution of sodium succinate (10 mmol, 1.6 g) was added to an aqueous solution of Cu(NO_3_)_2_·3H_2_O (10 mmol, 2.4 g) under stirring. A light-blue precipitate was formed. The precipitate was filtered and washed with water. The precipitate was dispersed in water and tetra­methyl­ethylenedi­amine (10 mmol, 1.2 g) was added giving a dark-blue solution. The solution was filtered. Single crystals were obtained on slow evaporation of the solution after one week.

## Refinement   

Crystal data, data collection and structure refinement details are summarized in Table 3 ▸. Carbon-bound H atoms were positioned geometrically and refined using a riding model, with C—H = 0.93, 0.96 and 0.97 Å with *U*_iso_(H) = 1.5*U*_eq_(C) for methyl H atoms and 1.2*U*_eq_(C) otherwise. The methyl groups were modelled as disordered over two torsional orientations. Water hydrogen-atom coordinates were refined, but *U*_iso_(H) was set to 1.5*U*_eq_(water O).

## Supplementary Material

Crystal structure: contains datablock(s) I. DOI: 10.1107/S2056989020007227/pk2626sup1.cif

Structure factors: contains datablock(s) I. DOI: 10.1107/S2056989020007227/pk2626Isup3.hkl

CCDC reference: 2006634

Additional supporting information:  crystallographic information; 3D view; checkCIF report

## Figures and Tables

**Table 1:** Selected geometric parameters (Å, °)

Cu1—O1	1.9639 (17)	O1—C7	1.275 (3)
Cu1—O3^i^	1.9958 (16)	O2—C7	1.236 (3)
Cu1—O4^i^	2.4315 (17)	O3—C10	1.273 (3)
Cu1—N1	2.024 (2)	O4—C10	1.239 (3)
Cu1—N2	2.038 (2)	N1—C1	1.459 (5)
			
O1—Cu1—O3^i^	89.80 (7)	O1—Cu1—N2	92.40 (8)
O1—Cu1—O4^i^	91.00 (7)	O3^i^—Cu1—N1	94.20 (8)
O1—Cu1—N1	167.77 (9)	O3^i^—Cu1—N2	165.06 (8)

Symmetry code: (i) 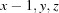.

**Table 2:** Hydrogen-bond geometry (Å, °)

*D*—H⋯*A*	*D*—H	H⋯*A*	*D*⋯*A*	*D*—H⋯*A*
O5—H5*H*⋯O4	0.79 (2)	1.98 (2)	2.763 (3)	169 (6)
O6—H6*G*⋯O5	0.82 (2)	1.88 (2)	2.694 (3)	175 (5)
O7—H7*A*⋯O2	0.82 (2)	1.96 (2)	2.774 (3)	172 (6)
O8—H8*C*⋯O3	0.83 (2)	2.04 (2)	2.869 (3)	173 (5)
O8—H8*D*⋯O7	0.83 (2)	1.93 (2)	2.733 (4)	165 (5)
O7—H7*B*⋯O6^ii^	0.81 (1)	1.97 (2)	2.763 (4)	167 (6)
O5—H5*G*⋯O1^iii^	0.80 (2)	2.00 (2)	2.799 (3)	176 (6)
O6—H6*H*⋯O8^iv^	0.84 (2)	2.01 (2)	2.803 (4)	157 (5)

Symmetry codes: (ii) 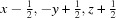; (iii) 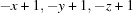; (iv) 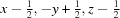.

**Table 3:** Experimental details

Crystal data	
Chemical formula	[Cu(C_4_H_4_O_4_)(C_6_H_16_N_2_)]·4H_2_O
*M*_r_	367.88
Crystal system, space group	Monoclinic, *P*2_1_/*n*
Temperature (K)	296
*a*, *b*, *c* (Å)	7.1195 (4), 12.3172 (6), 19.8590 (12)
β (°)	91.160 (5)
*V* (Å^3^)	1741.12 (17)
*Z*	4
Radiation type	Mo *K*α
μ (mm^−1^)	1.29
Crystal size (mm)	0.61 × 0.33 × 0.17

Data collection	
Diffractometer	Stoe IPDS 2
Absorption correction	Integration (*X-RED32*; Stoe & Cie, 2002 ▸)
*T*_min_, *T*_max_	0.645, 0.810
No. of measured, independent and observed [*I* > 2σ(*I*)] reflections	12278, 3427, 2864
*R*_int_	0.033
(sin θ/λ)_max_ (Å^−1^)	0.617

Refinement	
*R*[*F* ^2^ > 2σ(*F* ^2^)], *wR*(*F* ^2^), *S*	0.034, 0.091, 1.04
No. of reflections	3427
No. of parameters	233
No. of restraints	10
H-atom treatment	H atoms treated by a mixture of independent and constrained refinement
Δρ_max_, Δρ_min_ (e Å^−3^)	0.28, −0.26

Computer programs: *X-AREA* and *X-RED32* (Stoe & Cie, 2002 ▸), *SHELXT2017/1* (Sheldrick, 2015*a* ▸), *SHELXL2018/3* (Sheldrick, 2015*b* ▸) and *OLEX2* (Dolomanov *et al.*, 2009 ▸).

## supplementary crystallographic information

### Crystal data

**Table:**

[Cu(C_4_H_4_O_4_)(C_6_H_16_N_2_)]·4H_2_O	*F*(000) = 780
*M**_r_* = 367.88	*D*_x_ = 1.403 Mg m^−^^3^
Monoclinic, *P*2_1_/*n*	Mo *K*α radiation, λ = 0.71073 Å
*a* = 7.1195 (4) Å	Cell parameters from 19036 reflections
*b* = 12.3172 (6) Å	θ = 1.7–29.9°
*c* = 19.8590 (12) Å	µ = 1.29 mm^−^^1^
β = 91.160 (5)°	*T* = 296 K
*V* = 1741.12 (17) Å^3^	Stick, blue
*Z* = 4	0.61 × 0.33 × 0.17 mm

### Data collection

**Table:**

Stoe IPDS 2 diffractometer	2864 reflections with *I* > 2σ(*I*)
Detector resolution: 6.67 pixels mm^-1^	*R*_int_ = 0.033
rotation method scans	θ_max_ = 26.0°, θ_min_ = 2.0°
Absorption correction: integration (X-RED32; Stoe & Cie, 2002)	*h* = −8→8
*T*_min_ = 0.645, *T*_max_ = 0.810	*k* = −14→15
12278 measured reflections	*l* = −24→24
3427 independent reflections	

### Refinement

**Table:**

Refinement on *F*^2^	10 restraints
Least-squares matrix: full	Hydrogen site location: mixed
*R*[*F*^2^ > 2σ(*F*^2^)] = 0.034	H atoms treated by a mixture of independent and constrained refinement
*wR*(*F*^2^) = 0.091	*w* = 1/[σ^2^(*F*_o_^2^) + (0.056*P*)^2^ + 0.2662*P*] where *P* = (*F*_o_^2^ + 2*F*_c_^2^)/3
*S* = 1.04	(Δ/σ)_max_ = 0.001
3427 reflections	Δρ_max_ = 0.28 e Å^−^^3^
233 parameters	Δρ_min_ = −0.26 e Å^−^^3^

### Special details

**Table:**

Geometry. All esds (except the esd in the dihedral angle between two l.s. planes) are estimated using the full covariance matrix. The cell esds are taken into account individually in the estimation of esds in distances, angles and torsion angles; correlations between esds in cell parameters are only used when they are defined by crystal symmetry. An approximate (isotropic) treatment of cell esds is used for estimating esds involving l.s. planes.

### Fractional atomic coordinates and isotropic or equivalent isotropic displacement parameters (Å^2^)

**Table:**

	*x*	*y*	*z*	*U*_iso_*/*U*_eq_	Occ. (<1)
Cu1	0.21554 (4)	0.28617 (2)	0.62467 (2)	0.03940 (11)	
O1	0.3640 (2)	0.41083 (14)	0.59461 (8)	0.0471 (4)	
O2	0.5331 (3)	0.34615 (16)	0.67869 (10)	0.0581 (5)	
O3	1.0718 (2)	0.38395 (14)	0.68503 (8)	0.0457 (4)	
O4	0.9260 (3)	0.37395 (14)	0.58714 (8)	0.0512 (4)	
O7	0.6289 (4)	0.3189 (3)	0.81373 (13)	0.1013 (10)	
H7A	0.604 (7)	0.321 (5)	0.7735 (10)	0.152*	
H7B	0.535 (4)	0.302 (4)	0.834 (2)	0.152*	
O8	0.9959 (4)	0.3821 (3)	0.82634 (12)	0.0839 (7)	
H8C	1.023 (7)	0.387 (4)	0.7861 (12)	0.126*	
H8D	0.892 (4)	0.353 (4)	0.826 (3)	0.126*	
N1	0.1142 (4)	0.14824 (18)	0.66575 (12)	0.0592 (6)	
N2	0.3026 (3)	0.19183 (18)	0.54703 (11)	0.0545 (5)	
C1	0.2261 (7)	0.1220 (4)	0.7259 (2)	0.1078 (15)	
H1A	0.356188	0.117338	0.714512	0.129*	0.495 (18)
H1B	0.210105	0.177807	0.759095	0.129*	0.495 (18)
H1C	0.185516	0.053701	0.743793	0.129*	0.495 (18)
H1D	0.145018	0.115226	0.763755	0.129*	0.505 (18)
H1E	0.291101	0.054757	0.719171	0.129*	0.505 (18)
H1F	0.315690	0.178863	0.734474	0.129*	0.505 (18)
C2	−0.0839 (5)	0.1548 (3)	0.6849 (3)	0.1029 (15)	
H2A	−0.160188	0.172027	0.645981	0.124*	0.505 (18)
H2B	−0.122672	0.086297	0.703029	0.124*	0.505 (18)
H2C	−0.098082	0.210404	0.718331	0.124*	0.505 (18)
H2D	−0.093773	0.140458	0.732246	0.124*	0.495 (18)
H2E	−0.131289	0.226188	0.675199	0.124*	0.495 (18)
H2F	−0.155879	0.102082	0.659896	0.124*	0.495 (18)
C3	0.086 (3)	0.0712 (12)	0.6087 (8)	0.085 (4)	0.495 (18)
H3A	−0.018047	0.093743	0.579712	0.102*	0.495 (18)
H3B	0.062106	−0.001667	0.624964	0.102*	0.495 (18)
C3A	0.179 (2)	0.0582 (10)	0.6193 (8)	0.077 (3)	0.505 (18)
H3AA	0.302001	0.033292	0.633975	0.093*	0.505 (18)
H3AB	0.092507	−0.002668	0.622077	0.093*	0.505 (18)
C4	0.276 (3)	0.0761 (8)	0.5704 (6)	0.079 (3)	0.495 (18)
H4A	0.378964	0.054535	0.600078	0.095*	0.495 (18)
H4B	0.271645	0.027282	0.532075	0.095*	0.495 (18)
C4A	0.187 (2)	0.0950 (11)	0.5496 (7)	0.087 (4)	0.505 (18)
H4AA	0.239477	0.038474	0.521768	0.105*	0.505 (18)
H4AB	0.060916	0.111090	0.532601	0.105*	0.505 (18)
C5	0.2202 (9)	0.2343 (5)	0.4859 (2)	0.144 (3)	
H5A	0.086425	0.238681	0.490305	0.173*	0.495 (18)
H5B	0.269870	0.305336	0.477526	0.173*	0.495 (18)
H5C	0.249344	0.187114	0.449077	0.173*	0.495 (18)
H5D	0.317334	0.248739	0.454300	0.173*	0.505 (18)
H5E	0.133889	0.182085	0.467080	0.173*	0.505 (18)
H5F	0.154415	0.300307	0.495528	0.173*	0.505 (18)
C6	0.5040 (6)	0.1852 (4)	0.5403 (3)	0.1154 (17)	
H6A	0.559369	0.156878	0.581131	0.138*	0.505 (18)
H6B	0.533023	0.138122	0.503435	0.138*	0.505 (18)
H6C	0.553549	0.256345	0.531883	0.138*	0.505 (18)
H6D	0.537925	0.210685	0.496501	0.138*	0.495 (18)
H6E	0.564271	0.229441	0.574198	0.138*	0.495 (18)
H6F	0.543745	0.111219	0.545750	0.138*	0.495 (18)
C7	0.5046 (3)	0.41647 (19)	0.63531 (12)	0.0424 (5)	
C8	0.6324 (4)	0.51218 (19)	0.62714 (14)	0.0484 (6)	
H8A	0.566631	0.577064	0.641138	0.058*	
H8B	0.659682	0.520189	0.579733	0.058*	
C9	0.8161 (4)	0.5050 (2)	0.66639 (14)	0.0510 (6)	
H9A	0.882166	0.573443	0.662250	0.061*	
H9B	0.789224	0.494519	0.713636	0.061*	
C10	0.9432 (3)	0.41491 (19)	0.64394 (12)	0.0409 (5)	
O5	0.7915 (5)	0.3966 (2)	0.45643 (13)	0.1012 (10)	
H5G	0.751 (8)	0.454 (3)	0.443 (3)	0.152*	
H5H	0.827 (8)	0.399 (5)	0.4946 (13)	0.152*	
O6	0.8144 (4)	0.2050 (2)	0.39169 (17)	0.0906 (8)	
H6G	0.801 (7)	0.263 (3)	0.411 (2)	0.136*	
H6H	0.715 (5)	0.197 (4)	0.369 (2)	0.136*	

### Atomic displacement parameters (Å^2^)

**Table:**

	*U*^11^	*U*^22^	*U*^33^	*U*^12^	*U*^13^	*U*^23^
Cu1	0.03491 (16)	0.04272 (17)	0.04064 (16)	0.00251 (12)	0.00232 (10)	0.00379 (11)
O1	0.0359 (9)	0.0524 (9)	0.0529 (9)	0.0013 (7)	−0.0021 (7)	0.0085 (7)
O2	0.0532 (11)	0.0621 (11)	0.0589 (11)	−0.0009 (9)	−0.0042 (8)	0.0186 (9)
O3	0.0373 (9)	0.0555 (10)	0.0443 (9)	0.0048 (7)	−0.0012 (7)	−0.0012 (7)
O4	0.0481 (10)	0.0573 (10)	0.0482 (9)	0.0097 (8)	−0.0044 (7)	−0.0052 (8)
O7	0.0633 (15)	0.182 (3)	0.0581 (14)	−0.0182 (18)	−0.0042 (11)	0.0190 (17)
O8	0.0686 (15)	0.124 (2)	0.0586 (12)	−0.0156 (14)	−0.0081 (11)	0.0052 (14)
N1	0.0668 (15)	0.0447 (11)	0.0665 (14)	0.0005 (11)	0.0140 (11)	0.0083 (10)
N2	0.0558 (13)	0.0586 (13)	0.0494 (12)	0.0057 (10)	0.0066 (10)	−0.0055 (10)
C1	0.115 (3)	0.107 (3)	0.101 (3)	0.013 (3)	−0.002 (2)	0.059 (3)
C2	0.068 (2)	0.078 (2)	0.164 (4)	−0.0142 (19)	0.028 (2)	0.037 (3)
C3	0.101 (10)	0.061 (6)	0.095 (7)	−0.012 (7)	0.034 (8)	−0.005 (5)
C3A	0.076 (7)	0.045 (4)	0.112 (8)	0.000 (5)	0.020 (7)	0.004 (4)
C4	0.116 (10)	0.055 (4)	0.069 (6)	0.017 (5)	0.026 (6)	−0.002 (4)
C4A	0.096 (8)	0.066 (6)	0.100 (8)	−0.023 (5)	0.011 (6)	−0.031 (6)
C5	0.208 (6)	0.169 (5)	0.055 (2)	0.100 (5)	−0.035 (3)	−0.033 (3)
C6	0.067 (2)	0.148 (4)	0.132 (4)	0.014 (3)	0.026 (2)	−0.067 (3)
C7	0.0361 (12)	0.0459 (12)	0.0453 (12)	0.0079 (10)	0.0072 (9)	0.0023 (10)
C8	0.0395 (13)	0.0412 (12)	0.0646 (15)	0.0047 (10)	0.0023 (11)	0.0016 (11)
C9	0.0414 (13)	0.0450 (13)	0.0664 (16)	0.0019 (11)	−0.0021 (11)	−0.0103 (11)
C10	0.0337 (12)	0.0433 (11)	0.0459 (12)	−0.0035 (9)	0.0034 (9)	0.0006 (10)
O5	0.170 (3)	0.0614 (13)	0.0705 (15)	0.0345 (16)	−0.0484 (17)	−0.0091 (12)
O6	0.0686 (16)	0.0971 (19)	0.106 (2)	0.0074 (14)	0.0017 (13)	−0.0434 (15)

### Geometric parameters (Å, º)

**Table:**

Cu1—O1	1.9639 (17)	C3—H3A	0.9700
Cu1—O3^i^	1.9958 (16)	C3—H3B	0.9700
Cu1—O4^i^	2.4315 (17)	C3—C4	1.56 (2)
Cu1—N1	2.024 (2)	C3A—H3AA	0.9700
Cu1—N2	2.038 (2)	C3A—H3AB	0.9700
Cu1—C10^i^	2.540 (2)	C3A—C4A	1.46 (2)
O1—C7	1.275 (3)	C4—H4A	0.9700
O2—C7	1.236 (3)	C4—H4B	0.9700
O3—C10	1.273 (3)	C4A—H4AA	0.9700
O4—C10	1.239 (3)	C4A—H4AB	0.9700
O7—H7A	0.815 (19)	C5—H5A	0.9600
O7—H7B	0.812 (10)	C5—H5B	0.9600
O8—H8C	0.828 (19)	C5—H5C	0.9600
O8—H8D	0.827 (19)	C5—H5D	0.9600
N1—C1	1.459 (5)	C5—H5E	0.9600
N1—C2	1.470 (4)	C5—H5F	0.9600
N1—C3	1.489 (14)	C6—H6A	0.9600
N1—C3A	1.520 (14)	C6—H6B	0.9600
N2—C4	1.513 (10)	C6—H6C	0.9600
N2—C4A	1.453 (11)	C6—H6D	0.9600
N2—C5	1.436 (5)	C6—H6E	0.9600
N2—C6	1.445 (4)	C6—H6F	0.9600
C1—H1A	0.9600	C7—C8	1.500 (3)
C1—H1B	0.9600	C8—H8A	0.9700
C1—H1C	0.9600	C8—H8B	0.9700
C1—H1D	0.9600	C8—C9	1.512 (3)
C1—H1E	0.9600	C9—H9A	0.9700
C1—H1F	0.9600	C9—H9B	0.9700
C2—H2A	0.9600	C9—C10	1.504 (3)
C2—H2B	0.9600	O5—H5G	0.80 (2)
C2—H2C	0.9600	O5—H5H	0.794 (19)
C2—H2D	0.9600	O6—H6G	0.821 (19)
C2—H2E	0.9600	O6—H6H	0.835 (19)
C2—H2F	0.9600		
			
O1—Cu1—O3^i^	89.80 (7)	C4—C3—H3B	111.0
O1—Cu1—O4^i^	91.00 (7)	N1—C3A—H3AA	109.3
O1—Cu1—N1	167.77 (9)	N1—C3A—H3AB	109.3
O1—Cu1—N2	92.40 (8)	H3AA—C3A—H3AB	108.0
O1—Cu1—C10^i^	88.56 (7)	C4A—C3A—N1	111.5 (11)
O3^i^—Cu1—O4^i^	58.26 (6)	C4A—C3A—H3AA	109.3
O3^i^—Cu1—N1	94.20 (8)	C4A—C3A—H3AB	109.3
O3^i^—Cu1—N2	165.06 (8)	N2—C4—C3	107.6 (10)
O3^i^—Cu1—C10^i^	29.61 (7)	N2—C4—H4A	110.2
O4^i^—Cu1—C10^i^	28.77 (6)	N2—C4—H4B	110.2
N1—Cu1—O4^i^	100.93 (8)	C3—C4—H4A	110.2
N1—Cu1—N2	86.72 (9)	C3—C4—H4B	110.2
N1—Cu1—C10^i^	100.59 (9)	H4A—C4—H4B	108.5
N2—Cu1—O4^i^	106.90 (8)	N2—C4A—C3A	108.8 (11)
N2—Cu1—C10^i^	135.64 (9)	N2—C4A—H4AA	109.9
C7—O1—Cu1	105.67 (14)	N2—C4A—H4AB	109.9
C10—O3—Cu1^ii^	99.60 (14)	C3A—C4A—H4AA	109.9
C10—O4—Cu1^ii^	80.46 (14)	C3A—C4A—H4AB	109.9
H7A—O7—H7B	109 (2)	H4AA—C4A—H4AB	108.3
H8C—O8—H8D	105 (5)	N2—C5—H5A	109.5
C1—N1—Cu1	108.8 (2)	N2—C5—H5B	109.5
C1—N1—C2	108.1 (3)	N2—C5—H5C	109.5
C1—N1—C3	123.0 (8)	H5A—C5—H5B	109.5
C1—N1—C3A	99.7 (6)	H5A—C5—H5C	109.5
C2—N1—Cu1	114.2 (2)	H5B—C5—H5C	109.5
C2—N1—C3	96.8 (7)	H5D—C5—H5E	109.5
C2—N1—C3A	120.0 (6)	H5D—C5—H5F	109.5
C3—N1—Cu1	105.8 (5)	H5E—C5—H5F	109.5
C3A—N1—Cu1	104.7 (5)	N2—C6—H6A	109.5
C4—N2—Cu1	105.2 (4)	N2—C6—H6B	109.5
C4A—N2—Cu1	105.0 (5)	N2—C6—H6C	109.5
C5—N2—Cu1	107.9 (2)	H6A—C6—H6B	109.5
C5—N2—C4	123.4 (8)	H6A—C6—H6C	109.5
C5—N2—C4A	96.1 (8)	H6B—C6—H6C	109.5
C5—N2—C6	109.4 (4)	H6D—C6—H6E	109.5
C6—N2—Cu1	114.8 (2)	H6D—C6—H6F	109.5
C6—N2—C4	96.2 (7)	H6E—C6—H6F	109.5
C6—N2—C4A	121.6 (7)	O1—C7—C8	116.4 (2)
N1—C1—H1A	109.5	O2—C7—O1	121.3 (2)
N1—C1—H1B	109.5	O2—C7—C8	122.3 (2)
N1—C1—H1C	109.5	C7—C8—H8A	108.6
H1A—C1—H1B	109.5	C7—C8—H8B	108.6
H1A—C1—H1C	109.5	C7—C8—C9	114.7 (2)
H1B—C1—H1C	109.5	H8A—C8—H8B	107.6
H1D—C1—H1E	109.5	C9—C8—H8A	108.6
H1D—C1—H1F	109.5	C9—C8—H8B	108.6
H1E—C1—H1F	109.5	C8—C9—H9A	108.7
N1—C2—H2A	109.5	C8—C9—H9B	108.7
N1—C2—H2B	109.5	H9A—C9—H9B	107.6
N1—C2—H2C	109.5	C10—C9—C8	114.2 (2)
H2A—C2—H2B	109.5	C10—C9—H9A	108.7
H2A—C2—H2C	109.5	C10—C9—H9B	108.7
H2B—C2—H2C	109.5	O3—C10—Cu1^ii^	50.79 (11)
H2D—C2—H2E	109.5	O3—C10—C9	117.4 (2)
H2D—C2—H2F	109.5	O4—C10—Cu1^ii^	70.77 (13)
H2E—C2—H2F	109.5	O4—C10—O3	121.2 (2)
N1—C3—H3A	111.0	O4—C10—C9	121.4 (2)
N1—C3—H3B	111.0	C9—C10—Cu1^ii^	166.01 (17)
N1—C3—C4	104.0 (13)	H5G—O5—H5H	113 (5)
H3A—C3—H3B	109.0	H6G—O6—H6H	105 (3)
C4—C3—H3A	111.0		
			
Cu1—O1—C7—O2	5.8 (3)	N1—C3A—C4A—N2	54.0 (19)
Cu1—O1—C7—C8	−174.58 (16)	C1—N1—C3—C4	−76.7 (11)
Cu1^ii^—O3—C10—O4	7.5 (3)	C1—N1—C3A—C4A	−145.2 (12)
Cu1^ii^—O3—C10—C9	−171.00 (18)	C2—N1—C3—C4	166.6 (11)
Cu1^ii^—O4—C10—O3	−6.2 (2)	C2—N1—C3A—C4A	97.2 (12)
Cu1^ii^—O4—C10—C9	172.3 (2)	C5—N2—C4—C3	−84.0 (12)
Cu1—N1—C3—C4	49.0 (13)	C5—N2—C4A—C3A	−155.7 (13)
Cu1—N1—C3A—C4A	−32.7 (14)	C6—N2—C4—C3	157.9 (13)
Cu1—N2—C4—C3	40.1 (15)	C6—N2—C4A—C3A	87.1 (12)
Cu1—N2—C4A—C3A	−45.4 (15)	C7—C8—C9—C10	65.1 (3)
O1—C7—C8—C9	−168.5 (2)	C8—C9—C10—Cu1^ii^	169.4 (6)
O2—C7—C8—C9	11.1 (3)	C8—C9—C10—O3	−160.5 (2)
N1—C3—C4—N2	−60.5 (18)	C8—C9—C10—O4	20.9 (3)

Symmetry codes: (i) *x*−1, *y*, *z*; (ii) *x*+1, *y*, *z*.

### Hydrogen-bond geometry (Å, º)

**Table:**

*D*—H···*A*	*D*—H	H···*A*	*D*···*A*	*D*—H···*A*
O5—H5*H*···O4	0.79 (2)	1.98 (2)	2.763 (3)	169 (6)
O6—H6*G*···O5	0.82 (2)	1.88 (2)	2.694 (3)	175 (5)
O7—H7*A*···O2	0.82 (2)	1.96 (2)	2.774 (3)	172 (6)
O8—H8*C*···O3	0.83 (2)	2.04 (2)	2.869 (3)	173 (5)
O8—H8*D*···O7	0.83 (2)	1.93 (2)	2.733 (4)	165 (5)
O7—H7*B*···O6^iii^	0.81 (1)	1.97 (2)	2.763 (4)	167 (6)
O5—H5*G*···O1^iv^	0.80 (2)	2.00 (2)	2.799 (3)	176 (6)
O6—H6*H*···O8^v^	0.84 (2)	2.01 (2)	2.803 (4)	157 (5)

Symmetry codes: (iii) *x*−1/2, −*y*+1/2, *z*+1/2; (iv) −*x*+1, −*y*+1, −*z*+1; (v) *x*−1/2, −*y*+1/2, *z*−1/2.
